# Special Issue “New Challenges and Perspectives in Polycystic Ovary Syndrome”

**DOI:** 10.3390/ijms26062665

**Published:** 2025-03-15

**Authors:** Jim Parker, Pierre Hofstee

**Affiliations:** 1 School of Medicine, University of Wollongong, Wollongong 2500, Australia; pierre.hofstee@thewomens.org.au; 2 Department of Obstetrics and Gynaecology, The Royal Women’s Hospital, Melbourne 3052, Australia

Polycystic ovary syndrome (PCOS) is a complex multisystem metabolic and endocrine disorder that impacts health throughout the lifespan [1]. PCOS is increasingly being viewed as an evolutionary mismatch disorder that manifests after continued exposure to nutritional and environmental factors related to contemporary lifestyle [2,3]. In utero exposure to hormonal, metabolic or environmental factors may result in developmental programming of susceptible gene variants that predispose the offspring to develop PCOS when exposed to adverse postnatal conditions [4,5]. Neuroendocrine and immunometabolic homeostatic regulatory pathways that were adaptive in ancestral environments become maladaptive in modern society [2,6,7]. This conceptualization of the pathogenesis of PCOS is supported by evidence from evolutionary biology and research on the prevention and reversal of the biochemical, hormonal, and clinical features of PCOS following lifestyle-based interventions [1,2,8,9,10].

PCOS is usually diagnosed in adolescence due to the combination of oligomenorrhea and clinical or biochemical hyperandrogenism (HA) [1]. Adults are diagnosed with PCOS if they have at least two of the three diagnostic requirements, including oligomenorrhea, HA, polycystic ovary morphology on ultrasound, or elevated antimullerian hormone (AMH), after exclusion of other causes [11]. PCOS can be a progressive metabolic and hormonal condition that results in insulin resistance (IR), clinical obesity, type 2 diabetes, metabolic syndrome, metabolic-associated steatotic liver disease, cardiovascular disease, and endometrial cancer [12]. Individuals with PCOS can have endometrial dysfunction that contributes to an increased risk of pregnancy complications including miscarriage, implantation failure, preeclampsia, preterm birth, fetal growth restriction, stillbirth, and gestational diabetes [13]. In addition, those with PCOS have an increased risk of psychological morbidity and reduced quality of life.

Despite almost one hundred years of research and tens of thousands of publications, there still exists a significant gap in our understanding of the pathogenesis and pathophysiology of PCOS. A recent publication identified 150 priorities to guide future clinical research in PCOS [14]. This Special Issue provides an excellent collection of original research articles that address a broad range of well-aligned and relevant topics. These include the role of the microbiome, new biomarkers for IR, the role of epistasis or single-nucleotide polymorphism interactions, and the complex mechanisms contributing to estrogen signalling dysfunction in the ovaries of women with PCOS. The final article provides an excellent review of the relationship between maternal pathophysiology, endometrial dysfunction, and pregnancy complications.

The first research article by Varhegyi et al. explored the role of biomarkers for mitochondrial dysfunction, specifically growth differentiation factor-15 (GDF-15) and mitochondrial DNA (mtDNA) deletions, in women with IR and/or PCOS. IR is a core pathophysiological feature of PCOS, and the discovery of reliable mitochondrial biomarkers would be a useful adjunct to clinical management [15]. Mitochondrial dysfunction may be involved in and exacerbate the pathogenesis of IR-PCOS, through reduced mitochondrial membrane potential, ATP production, mitochondrial plasticity, and insulin-stimulated mitochondrial activity [16]. Concomitantly, these processes increase ROS production and lipid metabolites, further exacerbating IR.

Maternally inherited mtDNA serves as a significant biomarker, with deletions recognized as a sensitive indicator of mitochondrial dysfunction [17]. GDF-15 is a significant plasma mitochondrial biomarker and a stress-induced cytokine member of the transforming growth factor beta superfamily. GDF-15 is known to be elevated in T2DM, positively correlates with IR, and is linked to several pathological conditions [18]. This study was the first to investigate GDF-15 in women with PCOS and aimed to delineate the correlation between GDF-15 and IR, with or without PCOS. Additionally, the authors further investigated correlations between GDF-15 and dosage requirements for metformin, and mtDNA deletion effects on GDF-15 in the study population. Although no association between the GDF-15 biomarker and mtDNA deletion was detected in the studied patient group, GDF-15 levels increased with age, BMI, and daily dose of metformin, and plasma levels were associated with reactive hyperinsulinemia following an oral glucose tolerance test.

Women with IR or IR-PCOS had a higher prevalence of mtDNA deletions and mitochondrial dysfunction, which was more significant in the IR subgroup. The authors were unable to distinguish whether the observed results were a consequence of high plasma GDF-15 levels or mitochondrial dysfunction. They concluded that the presence of both may be a consequence of carbohydrate metabolism disorders in patients and may predict accelerated aging. Finally, though mitochondrial dysfunction may have contributed to disease development at a significantly higher rate in the IR-only subgroup than in patients with IR-PCOS, GDF-15 plasma levels may be related to the severity of IR.

Diet-induced gastrointestinal (GI) dysbiosis has been hypothesized to play a significant role in the pathogenesis of PCOS since the seminal paper of Tremellen and Pearce in 2012 [19]. The initial theory proposed that a high-sugar, high-fat, low-fibre diet caused adverse changes in the GI microbiome that impaired protective mucous and activated the zonulin pathway, thereby increasing GI permeability and translocation of lipopolysaccharide (LPS) endotoxins into the submucosa and circulation. LPS binds with toll-like receptor-4 on macrophages and activates the production of inflammatory cytokines via the nuclear factor kappa B pathway. Submucosal macrophages enter the circulation and initiate low-grade inflammatory changes in a variety of organs and tissues throughout the body. Both poor-quality diet and low-grade inflammation contribute to the development of IR and the subsequent clinical, biochemical, and hormonal changes observed in individuals with PCOS. A subsequent review of 31 studies that specifically investigated components of this hypothesis found convincing evidence that women with PCOS have reduced species richness and microbial imbalance, and provided support for the proposed pathophysiological mechanism [20]. Recently, a number of other mechanisms have been proposed including the role of bile acids, choline, indole, the gut–brain axis, and short-chain fatty acids (SCFAs) [21]. SCFAs play a crucial role in maintaining the integrity of the mucosal barrier, act as signalling molecules within the gut ecosystem, and have multiple beneficial systemic metabolic and immune effects such as improving insulin sensitivity and overall metabolic health. The second paper by Kukaev et al. is a prospective study that aimed to investigate the effect of metformin therapy on the composition of the intestinal flora and production of SCFA metabolites in women with PCOS.

Metformin is commonly used as a treatment for metabolic and reproductive symptoms of PCOS and is endorsed by the 2023 International Guidelines for the management of PCOS [1]. The study investigated SCFA levels in fecal and blood samples in 69 women with PCOS before and after 6 months’ treatment with metformin at 1500 mg per day and compared the results to 18 healthy controls. The results showed that women with PCOS had reduced microbial diversity before treatment, in line with previous studies and reviews (Figure 1) [20,21]. Following treatment with metformin, women with PCOS had an increase in symbiotic gut bacteria such as A *muciniphila* and a decrease in opportunistic pathogens such as C *perfringens* and C *difficile*. Treatment with metformin demonstrated a selective effect on SCFA levels in feces, significantly reducing acetic acid levels. Serum levels of SCFA persistently decreased but did not reach statistical significance compared to controls. The authors concluded that the impact of metformin may be more pronounced in the GI tract than in serum.

In addition to the favorable effect on microbiome composition and SCFAs, metformin treatment also reduced the frequency of IR, improved markers of chronic inflammation, resulted in significant positive changes in body composition, and improved menstrual cycle regularity in over 50% of study participants. Women who experienced complete cycle regulation were found to have a significant increase in the abundance of beneficial bacteria compared to those with a partial response to therapy. Interestingly, the identified species are known to be involved in the production of bile acids, which have previously been hypothesized to have a role in the pathophysiology of PCOS [21]. This study adds to existing evidence that the gut microbiome plays a pivotal role in the pathogenesis of PCOS. Further attention is drawn to the role of SCFAs and bile acids in the pathophysiology of PCOS, and the findings provide greater insight into the role of personalized treatment strategies, including dietary modification, symbiotic supplementation, and metformin therapy.

Two studies explored different aspects of the molecular mechanisms and genetics of PCOS. Alarcon-Granados et al. used a machine learning approach to investigate the effect of epistasis, or gene interactions, between 27 single-nucleotide polymorphisms identified as risk candidates in women with PCOS. They reported both synergistic and redundant gene effects, suggesting that gene interactions contribute to PCOS susceptibility and paving the way for future studies to investigate the impact of epistasis on the phenotypic features of PCOS. Marie et al. analyzed the transcriptome abundance of 16 genes in granulosa cells, finding that androgen and progesterone receptor expression was significantly increased, while that of the progesterone synthesis enzymes (*CYP11A1* and *HSD3B2*) was downregulated. Using liquid chromatography–tandem mass spectrometry, they examined steroid hormone concentration in follicular fluid of polycystic ovaries, confirming that PCOS follicular fluid was hyperandrogenic and lower in estradiol [22,23]. However, finding that estrogen biosynthesis was normal, the authors suggested that the reduced follicular fluid estradiol levels were due to increased degradation rather than reduced aromatase activity or estradiol biosynthesis, as is commonly believed [24,25,26]. Further investigations are required to confirm these findings and assess whether this mechanism contributes to estrogen dysregulation in other estrogen-responsive tissues such as endometrial, breast, hypothalamus, or pituitary tissue.

The final article by Matsuyama et al. is a review that explores the impact of core maternal pathophysiological states (IR, inflammation, hyperandrogenism) on endometrial function related to implantation and decidualization in women with PCOS. There has been increasing interest in the impact of pre-existing metabolic, hormonal, and immune dysfunctions and their adverse effects on fertility and pregnancy outcomes in women with PCOS [13]. This review addresses some of the changes that are known to occur in hormonal, metabolic, and cellular signalling pathways and their impact on reproduction.

Estrogens, progesterone, and androgens have important roles in endometrial physiology and contribute to the development of a suitable microenvironment for implantation [27]. HA alters the LH/FSH ratio, leading to hormonal and cytokine changes such as decreased progesterone and leukemia inhibitory factor, which inhibit decidualization of endometrial stromal cells and have a negative effect on implantation [28,29]. Elevated testosterone levels result in decreased Claudin 4, Claudin 1, and Occludin, which compromises tight junctions between endometrial epithelial cells, possibly contributing to impaired embryo attachment and implantation [30]. Women with PCOS have decreased serum 17-beta-estradiol levels in the lower normal range but may have higher levels in the endometrium [31,32]. Estrogen receptor 1 levels are significantly increased in the endometrium of both women with PCOS and animal models. Oligo-ovulation changes the estrogen-to-progesterone balance, alters endometrial hormone and receptor concentrations, upregulates cellular proliferation proteins (Ki-67), and disrupts cellular signalling pathways (LIF-STAT3) (Figure 2) [33].

Insulin resistance impairs glucose metabolism within the endometrium and promotes progesterone resistance and impaired decidualization [27]. Hyperinsulinemia can stimulate increased androgen production by ovarian theca cells and reduce aromatization of testosterone to estradiol in granulosa cells [15,34]. The increased androgen production creates a positive feedback loop that exacerbates hormonal and metabolic dysfunction. The authors discuss the relationship between persistent low-grade inflammation and metabolic and reproductive function, and the link with inflammation in the endometrium of women with PCOS. They provide a detailed review of the relationship between the pathophysiologic features of PCOS and reproductive outcomes and reinforce the evidence-based guideline recommendation that lifestyle modification should be the primary treatment to improve metabolic and reproductive outcomes [1]. This review explores new mechanistic research that connects maternal health to pregnancy outcomes and aligns with the theme of this Special Issue.

In summary, this Special Issue highlights research investigating new challenges and perspectives in PCOS. It includes research on new biomarkers of IR, the effect of metformin on the GI microbiome, novel findings in ovarian estrogen signalling, and previously unexplored interactions between PCOS susceptibility polymorphisms (epistasis). These data should provide practical information to enhance future developments in a number of important areas of PCOS research. In particular, this could include research on the predictive value of mitochondrial markers of IR, the therapeutic effect of metformin on the microbiome, the efficacy of fecal microbial transplant, the influence of epistasis on the pathophysiology of PCOS, and the role of newly identified estrogen signalling changes in the ovary, endometrium, hypothalamus, and pituitary. Clinicians and researchers can gain valuable insights into the pathophysiology of PCOS from this collection of papers.

## Figures and Tables

**Figure 1:**
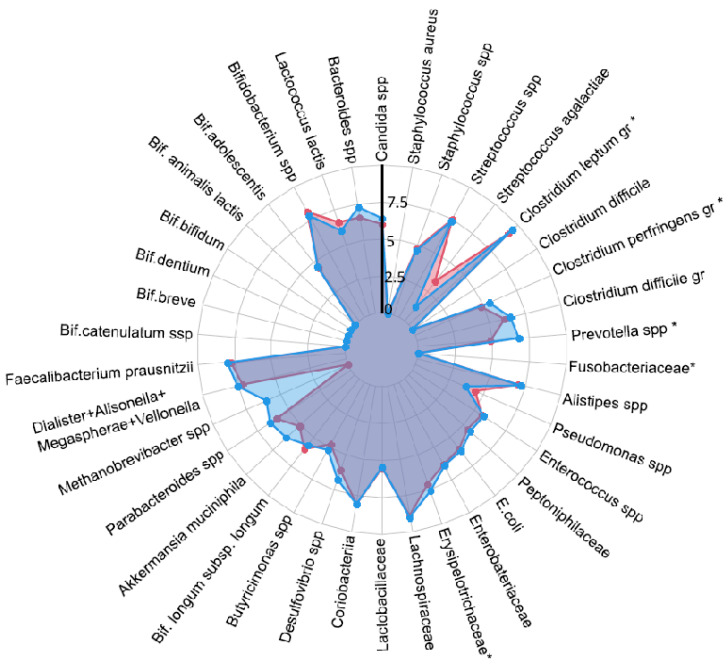
The influence of PCOS on gut microbiota before treatment, presented as a radar chart (logarithmic scale) with median values of the number of colony-forming units: PCOS (red color, n = 69) and control group (blue color, n = 18). The data represent the average estimate of the log10 of fecal real-time polymerase chain reaction (PCR) target genetic amplicon copy numbers in 1 g of feces. Statistically significant alterations (*p* < 0.05) are indicated by an asterisk (*). Adopted from Kukaev et al.

**Figure 2:**
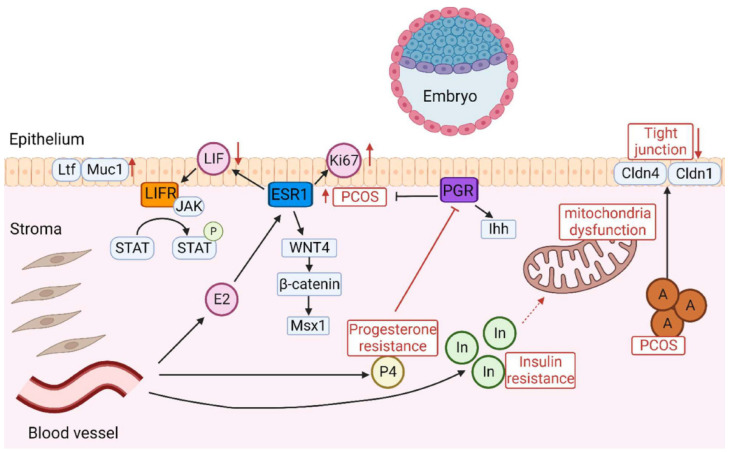
Diagram of the intricate signalling pathways involved in embryo implantation. In PCOS, abnormal expression of ESR1 increases Ki-67 expression in the endometrial epithelium and stroma, leading to decreased endometrial receptivity. LIF triggers the activation of JAK, which in turn activates signal transducer and activator of STAT3 through phosphorylation. The role of the LIFSTAT3 pathway is characterized by inactivation in PCOS, which leads to the production of Ltf and Muc1, suggesting that the endometrium is in a non-receptive state. The dysregulation of the Wnt/β-catenin/Msx1 pathway impairs endometrial receptivity in PCOS, and Msx1 is increased. Insulin resistance contributes to progesterone resistance and exacerbates issues with endometrial functionality. The abnormal expression of Ihh signalling in PCOS is depicted as a key factor affecting endometrial receptivity. Elevated androgen levels impact the integrity of tight junctions in the endometrial epithelium by decreasing Cldn4 and Cldn1. Insulin resistance impairs endometrial receptivity in PCOS, leading to mitochondrial dysfunction. A: androgen; P4: progesterone; E2: estrogen; In: insulin; P: phosphorylation. Adopted from Matsuyama et al.

## References

[1] Teede H.J., Tay C.T., Laven J., Dokras A., Moran L., Piltonen T., Costello M., Boivin J., Redman L., Boyle J. (2023). International evidence-based guideline for the assessment and management of polycystic ovary syndrome 2023. Natl. Health Med. Res. Counc..

[2] Parker J., O’Brien C., Hawrelak J., Gersh F.L. (2022). Polycystic Ovary Syndrome: An Evolutionary Adaptation to Lifestyle and the Environment. Int. J. Environ. Res. Public Health.

[3] Dumesic D.A., Abbott D.H., Chazenbalk G.D., Scholar G. (2023). An Evolutionary Model for the Ancient Origins of Polycystic Ovary Syndrome. J. Clin. Med..

[4] Stener-Victorin E., Deng Q. (2025). Epigenetic inheritance of PCOS by developmental programming and germline transmission. Trends Endocrinol. Metab..

[5] Shaw L.M.A., Elton S. (2008). Polycystic ovary syndrome: A transgenerational evolutionary adaptation. BJOG Int. J. Obstet. Gynaecol..

[6] Charifson M.A., Trumble B.C. (2019). Evolutionary origins of polycystic ovary syndrome: An environmental mismatch disorder. Evol. Med. Public Health.

[7] Dumesic D.A., Padmanabhan V., Chazenbalk G.D., Abbott D.H. (2022). Polycystic ovary syndrome as a plausible evolutionary outcome of metabolic adaptation. Reprod. Biol. Endocrinol..

[8] Benton M.L. (2021). The influence of evolutionary history on human health and disease. Nat. Rev. Genet..

[9] Fay J.C. (2013). Disease consequences of human adaptation. Appl. Transl. Genom..

[10] Tsatsoulis A., Mantzaris M.D., Bellou S., Andrikoula M. (2013). Insulin resistance: An adaptive mechanism becomes maladaptive in the current environment—An evolutionary perspective. Metabolism.

[11] Joham A.E., Tay C.T., Laven J., Louwers Y.V., Azziz R. (2025). Approach to the Patient: Diagnostic Challenges in the Workup for Polycystic Ovary Syndrome. J. Clin. Endocrinol. Metab..

[12] Hoeger K.M., Dokras A., Piltonen T. (2021). Update on PCOS: Consequences, Challenges, and Guiding Treatment. J. Clin. Endocrinol. Metab..

[13] Parker J., Hofstee P., Brennecke S. (2024). Prevention of Pregnancy Complications Using a Multimodal Lifestyle, Screening, and Medical Model. J. Clin. Med..

[14] Teede H.J., Gibson M., Laven J., Dokras A., Moran L.J., Piltonin T., Costello M., Mousa A., Joham A.E., Tay C.T. (2024). International PCOS guideline clinical research priorities roadmap: A co-designed approach aligned with end-user priorities in a neglected women’s health condition. Eclinicalmedicine.

[15] Parker J. (2023). Pathophysiological Effects of Contemporary Lifestyle on Evolutionary-Conserved Survival Mechanisms in Polycystic Ovary Syndrome. Life.

[16] Siemers K.M., Klein A.K., Baack M.L. (2023). Mitochondrial Dysfunction in PCOS: Insights into Reproductive Organ Pathophysiology. Int. J. Mol. Sci..

[17] Boenzi S., Diodato D. (2018). Biomarkers for mitochondrial energy metabolism diseases. Essays Biochem..

[18] Pence B.D. (2022). Growth Differentiation Factor-15 in Immunity and Aging. Front. Aging.

[19] Tremellen K., Pearce K. (2012). Dysbiosis of Gut Microbiota (DOGMA)-A novel theory for the development of Polycystic Ovarian Syndrome. Med. Hypotheses.

[20] Parker J., O’Brien C., Hawrelak J. (2021). A review of the role of gastrointestinal dysbiosis in thepathogenesis of polycystic ovary syndrome. J. Obstet. Gynecol. Res..

[21] Guo J., Shao J., Yang Y., Niu X., Liao J., Zhao Q., Wang D., Li S., Hu J. (2022). Gut Microbiota in Patients with Polycystic Ovary Syndrome: A Systematic Review. Reprod. Sci..

[22] Naessen T., Kushnir M.M., Chaika A., Nosenko J., Mogilevkina I., Rockwood A.L., Carlstrom K., Bergquist J., Kirilovas D. (2010). Steroid profiles in ovarian follicular fluid in women with and without polycystic ovary syndrome, analyzed by liquid chromatography-tandem mass spectrometry. Fertil. Steril..

[23] Yang Z., Zhou W., Zhou C., Zhou Y., Liu X., Ding G., Hu Y., Pan J., Sheng J., Jin L. (2021). Steroid metabolome profiling of follicular fluid in normo- and hyperandrogenic women with polycystic ovary syndrome. J. Steroid Biochem. Mol. Biol..

[24] Jakimiuk A.J., Weitsman S.R., Brzechffa P.R., Magoffin D.A. (1998). Aromatase mRNA expression in individual follicles from polycystic ovaries. Mol. Hum. Reprod..

[25] Panghiyangani R., Soeharso P., Pujianto A., Suryandari D., Wiweko B., Kurniati M., Pujianto D.A. (2020). CYP19A1 gene expression in patients with polycystic ovarian syndrome. J. Hum. Reprod. Sci..

[26] Narayana S., Ananad C., Kumari N.S., Sonkusere S., Babu S.V.S. (2023). Impact of Aromatase Enzyme and its Altered Regulation on Polycystic Ovary Syndrome (PCOS): A Key Factor in Pathogenesis of PCOS. Indian J. Med. Spec..

[27] Xue Z., Li J., Feng J., Han H., Zhao J., Zhang J., Han Y., Wu X., Zhang Y. (2021). Research Progress on the Mechanism Between Polycystic Ovary Syndrome and Abnormal Endometrium. Front. Physiol..

[28] Ali R., Ahmed T., Gul H., Rehman R. (2023). An interplay of Progesterone, Leukemia Inhibitor Factor and Interleukin-6 in the window of implantation; Impact on fertility. Cytokine.

[29] Yusuf M., Amri M.F., Ugusman A., Hamid A.A., Wahab N.A., Mokhtar M.H. (2023). Hyperandrogenism and Its Possible Effects on Endometrial Receptivity: A Review. Int. J. Mol. Sci..

[30] Mokhtar M.H., Giriabu N., Salleh N. (2020). Testosterone Reduces Tight Junction Complexity and Down-regulates Expression of Claudin-4 and Occludin in the Endometrium in Ovariectomized, Sex-steroid Replacement Rats. Vivo.

[31] Li S., Song Z., Song M., Qin J., Zhao M., Yang Z. (2016). Impaired receptivity and decidualization in DHEA-induced PCOS mice. Sci. Rep..

[32] Lindheim L., Bashir M., Munzker J., Trummer C., Zachhuber V., Leber B., Horvath A., Pieber T.R., Gorkiewicz G., Stadlbauer V. (2017). Alterations in Gut Microbiome Composition and Barrier Function Are Associated with Reproductive and Metabolic Defects in Women with Polycystic Ovary Syndrome (PCOS): A Pilot Study. PLoS ONE.

[33] Sun X., Bartos A., Whitsett J.A., Dey S.K. (2013). Uterine Deletion of Gp130 or Stat3 Shows Implantation Failure with Increased Estrogenic Responses. Mol. Endocrinol..

[34] Patel S. (2017). Disruption of aromatase homeostasis as the cause of a multiplicity of ailments: A comprehensive review. J. Steroid Biochem. Mol. Biol..

